# The Lack of Ad Hoc Neuropsychological Assessment in Adults with Neurofibromatosis: A Systematic Review

**DOI:** 10.3390/jcm13051432

**Published:** 2024-03-01

**Authors:** Giuseppa Maresca, Carmen Bonanno, Isabella Veneziani, Viviana Lo Buono, Desirèe Latella, Angelo Quartarone, Silvia Marino, Caterina Formica

**Affiliations:** 1 IRCCS Centro Neurolesi Bonino Pulejo, S.S. 113-Via Palermo, C.da Casazza, 98124 Messina, Italy; giusy.maresca@irccsme.it (G.M.); carmen.bonanno@irccsme.it (C.B.); viviana.lobuono@irccsme.it (V.L.B.); angelo.quartarone@irccsme.it (A.Q.); silvia.marino@irccsme.it (S.M.); katia.formica@irccsme.it (C.F.); 2 Department of Nervous System and Behavioural Sciences, Psychology Section, University of Pavia, Piazza Botta, 11, 27100 Pavia, Italy; isabellamaria.veneziani01@universitadipavia.it

**Keywords:** neurofibromatosis Type 1, neuropsychological assessment, cognitive impairment, neurorehabilitation, visuospatial functions, executive functions

## Abstract

**Background:** Neurofibromatosis Type 1 (NF1) is a genetic autosomal dominant disorder that affects both the central and peripheral nervous systems. Children and adolescents with NF1 commonly experience neuropsychological, motor, and behavioral deficits. The cognitive profile hallmark of this disorder includes visuospatial and executive function impairments. These cognitive disorders may persist into adulthood. This study aims to analyze previous research studies that have described cognitive dysfunctions in adults with NF1. The purpose of this analysis is to review the neuropsychological and psychological assessment methods used. **Methods:** A total of 327 articles were identified based on the search terms in their titles and abstracts. The evaluation was conducted by scrutinizing each article’s title, abstract, and text. **Results:** Only 16 articles were found to be eligible for inclusion based on the pre-defined criteria. The selected studies primarily focus on the development of diagnostic protocols for individuals with NF1. **Conclusions:** The management of NF1 disease requires a multidisciplinary approach to address symptoms, preserve neurological functions, and ensure the best possible quality of life. However, cognitive impairment can negatively affect psychological well-being. This study suggested that cognitive functions in NF1 patients were not tested using specific measures, but rather were evaluated through intelligence scales. Additionally, the findings revealed that there is no standardized neuropsychological assessment for adults with NF1. To address this gap, it would be helpful to create a specific neuropsychological battery to study cognitive function in NF1 patients during clinical studies. This battery could also serve as a tool to design models for cognitive rehabilitation by using reliable and sensitive measures of cognitive outcomes.

## 1. Introduction

Neurofibromatosis Type 1 (NF1) (MIM number 162200), also known as von Recklinghausen’s disease, is a genetic disorder that affects both the central and peripheral nervous systems [1]. The main features of the disease are: café-au-lait spots of the skin, Lisch nodules in the eyes, and fibromatous tumors of the skin. It is an autosomal dominant disorder, with a prevalence of 1 in 2500–3500 cases that are usually diagnosed in early childhood. NF1 is hereditary in approximately half of the cases, with 80% paternal transmission [2,3]. It is characterized by the presence of benign or malignant tumors that involve the central and peripheral nerves [4]. Peripheral nerve tumors, specifically neurofibromas, are more common in NF1. These tumors consist of an overgrowth of nerve tissue, blood vessels, fibers, and other types of cells such as Schwann cells, fibroblasts, neural cells, and mast cells [5,6,7]. Most manifestations of NF1 occur in childhood and adolescence, stages in which a certain rate of comorbidity such as attention deficit hyperactivity disorder (ADHD), learning disabilities, and autism spectrum are detected [8,9]. Children and adolescents with NF1 tend to develop common neuropsychological, motor, and behavioral deficits [10,11,12]. Thirty to sixty-five percent of children with NF1 showed learning problems, such as writing, language, reading, comprehension, spelling, and mathematics. A high frequency of sustained and divided attention processes in childhood was demonstrated [13]. The wide spectrum of attention impairment required a major focus during the basic screening. Furthermore, some studies have described executive dysfunction, visuospatial abilities alterations, and a lower IQ range (<70) in 4% to 8% of NF1 patients [14]. A recent meta-analysis described executive function deficits related to working memory, self-shifting, planning, and problem-solving and social competence [15,16]. Verbal and non-verbal cognitive deficits are common in children and adolescents with NF1, and visuospatial and executive functions represent the cognitive profile hallmark of this disorder.

### 1.1. State of Art of Neuroimaging Aspects

Neuroimaging studies demonstrated morphological abnormalities in T2-hyperintensities at the subcortical level, with the basal ganglia, cerebellum, thalamus, and hippocampus being the most involved cerebral structures [17,18]. A correlation between cognitive dysfunction and the number and location of T2-hyperintensities was demonstrated, particularly in the cerebellum and the thalamus [18]. Some studies have highlighted a correlation between cognitive dysfunction and T2-hyperintensities in subcortical areas [17,19,20]. The best predictor of cognitive dysfunction in adulthood was the presence of T2-hyperintensities in childhood, rather than current lesion status. There is a limited time window (<18 years) in which the presence of T2-hyperintensities can be used as biologic markers of cognitive dysfunction [17]. A relationship between cerebellum abnormalities and a compromised cognitive profile in NF1 patients, especially regarding visuospatial and language abilities, has been shown in some studies [18]. Preliminary investigations in this field could be useful to determine the brain biomarkers involved in screening and detecting a clear cognitive profile starting from neuroimaging techniques.

### 1.2. State of Art of Cognitive Aspects

Cognitive disorders that emerge in childhood may persist into adulthood. For this reason, it could be useful to provide and to structure a tailored neuropsychological and psychosocial assessment for these patients. Only a few studies on NF1 adults focused on cognitive status [14,15,20], while no reports about the cognitive progression in this disorder and neuropsychological assessment for the definition of their cognitive profile are available. In the absence of a clear diagnostic flow chart for adult patients with NF1 with neurocognitive dysfunctions, the aim of this systematic review is to identify and analyze studies that have described cognitive dysfunctions in adult patients with NF1 to review the neuropsychological and psychological assessment methods used for these patients.

## 2. Materials and Methods

**Figure 1:**
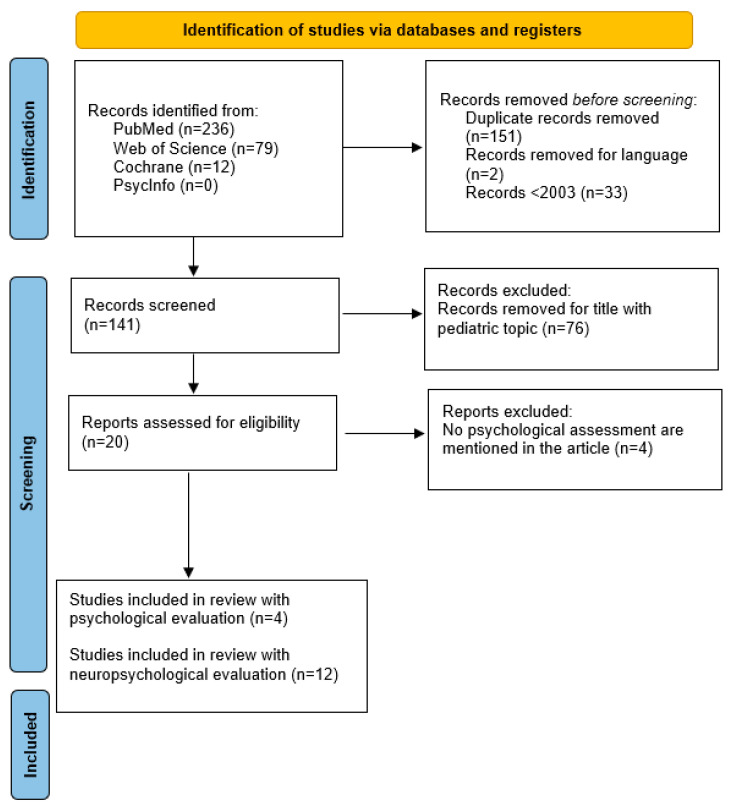
Prisma flow diagram for research strategy.

This systematic review was conducted and reported in accordance with the Preferred Reporting Items for Systematic Review and Meta-Analyses (PRISMA) (Figure 1). The systematic review was registered in OSF (identification number: doi:10.17605/OSF.IO/G5BHJ).

### 2.1. Search Strategy

Articles were selected from research databases: PubMed, Cochrane, Web of Science, and PsycInfo using the following search terms: (“neurofibromatoses” [MeSH Terms] OR “neurofibromatoses” [All Fields] OR “neurofibromatosis” [All Fields]) AND ((“psychologic” [All Fields] OR “psychological” [All Fields] OR “psychologically” [All Fields] OR “psychologization” [All Fields] OR “psychologized” [All Fields] OR “psychologizing” [All Fields]) AND (“assess” [All Fields] OR “assessed” [All Fields] OR “assessement” [All Fields] OR “assesses” [All Fields] OR “assessing” [All Fields] OR “assessment” [All Fields] OR “assessment s” [All Fields] OR “assessments” [All Fields])) AND (“neuropsychological tests” [MeSH Terms] OR (“neuropsychological” [All Fields] AND “tests” [All Fields]) OR “neuropsychological tests” [All Fields] OR (“neuropsychological” [All Fields] AND “assessment” [All Fields]) OR “neuropsychological assessment” [All Fields]) AND (“adult” [MeSH Terms] OR “adult” [All Fields] OR “adults” [All Fields] OR “adults” [All Fields]).

The search was limited to articles in English published in the past 20 years dealing with human subjects. Inclusion criteria: (i) original English articles that enrolled human subjects; (ii) experiments that include cognitive and psychological evaluation in adults; (iii) articles in the English language only; (iv) studies that included adults and young adults. Exclusion criteria: (i) studies that include a pediatric population; (ii) studies before 2003; (iii) reviews about NF1; (iv) duplicated studies.

### 2.2. Study Selection

A total of 327 articles were identified through database searches of PubMed, Cochrane, Web of Science, and PsycInfo. One hundred and fifty-one duplicated articles were deleted; 33 articles before 2003; 76 studies with pediatric topic in the title were deleted; 2 studies were removed for language; 45 studies for abstract (Figure 1). In this systematic review, we considered a total of 16 articles about neuropsychological and psychological aspects and assessment, respectively. Neuropsychological evaluation allows for an in-depth analysis of cognitive deficits associated with brain dysfunction and is useful for diagnosis according to some diagnostic criteria for neurological disorders [21]. Neuropsychological assessment in adults as a screening method is often used in differential diagnosis, often to distinguish between psychiatric and neurogenetic conditions, between different neurological conditions [22,23], and to identify the possible anatomical localization of the disorder [24]. Lezak, Howieson, and Loring (2004) argued that neuropsychological assessments are often used for: diagnosis, patient care, treatment planning, treatment evaluation, research, and forensics [22].

The study selection was conducted by two authors (CF and GM) who extracted data independently. Any disagreements were solved by discussion and by consulting a third author (DL). The methodological quality of the included articles was assessed according to the Newcastle–Ottawa Scale (NOS). Its content validity and inter-rater reliability have been established [25]. The NOS gives predefined criteria as following: selection (representativeness, selection of controls, ascertainment of exposure, no asthma at start of study); comparability (confounding); and outcome (assessment of outcome, length, and adequacy of follow-up) (Table 1). Each article was assessed by at least three authors independently. In case of disagreement, the other two authors were consulted. Data were extracted from the full-text article. Study selection was performed independently by two authors (CF and GM, who extracted data). If essential data were lacking in the original studies, their authors were contacted. All 16 studies were evaluated using the adapted NOS scale (Table 1), most of which obtained 4 points or more, indicating a moderate to good quality. Only two studies [26,27] obtained a lower value score.

## 3. Results

The present systematic review revealed moderate impairments in the majority of cognitive domains in patients with NF1. We included a total of 12 studies that investigate neuropsychological profiles [26,28,29,30,31,32,33,34,35,36,37,38]; 4 studies about behavioral and psychological constructs that included mood disorders and mental and physical health in terms of of Quality of Life (QoL) status [27,39,40,41]. The majority of these studies used intelligence scales to investigate IQ levels [28,29,30,31,35,36,38]. Few studies investigated other specific cognitive domains such as language [30,32,38], executive functions [30,32,36,38], and memory and visuospatial attention [28,29,30,32,38]. Finally, some authors described mood disorders [34,41], QoL [34,39,40,41], and behavioral aspects [36] (Table 2). In general, we found that almost all patients with NF1 have cognitive deficits, particularly of executive functions [15] and attentional and visuospatial processes [18] that represented the hallmark features. It is not yet clear whether these symptoms tend to worsen as the disease progresses. We found a study that used a neuropsychological assessment typical of neurological patients [31]. The studies that we analyzed reported a neuropsychological investigation mainly for the definition of IQ by using the Wechsler Adult Intelligence Scale (WAIS) for adult patients and the Wechsler Intelligence Scale for Children (WISC) for adolescents until 17 years old [28,29,30,31,35,36,38,41]. Only a few studies used different tests to examine memory, attention, and language deficits [29,30,32,38]. This suggests a non-homogeneous choice in the use of tests, probably due to the different objectives of the studies examined. For instance, Schutze et al. [30] opted to use tests to evaluate intelligence, attention, memory, and fine motor skills, employing a battery that included WAIS, the Rey–Osterrieth Complex Figure Test (ROCF) for visuospatial abilities, and the Brazilian–Portuguese version of the Rey Auditory–Verbal Learning Test (RAVLT). These tests were used for both children over the age of seven and adults. Another study evaluated visual skills, attention, executive function, and verbal ability, utilizing tests such as JLO and the Visual Form Discrimination test (VISFM), Visual-Motor Integration (VMI), the Booklet Category Test (CATEG), a non-verbal figure cancellation test (FIGCAN), and the Controlled Oral Word Association (COWA) [32]. The authors confirmed that the tests selected were previously used in literature studies to evaluate children with NF1 and were adapted for the participants of this study, who were 18 years old [32]. Some studies only used the WAIS for intelligence level without any other cognitive detailed tests [28,29,31,35]. Miguel et al. is the only study that employed a complete neuropsychological battery for an adult with NF1, although described in a case report only [38]. Two studies did not use a specific neuropsychological assessment [27,33]. The most commonly used test to assess behavior, mood, and quality of life is the World Health Organization QoL (WHOQOL) [39,40]. However, some authors used this test to evaluate the flexibility and usability of a videoconference program [39,40]. Lester et al. [41] found that suicidal ideation is prevalent in patients with NF1 who have emotional disorders and that depression and poor psychological QoL increase the risk of suicide.

## 4. Discussion

The purpose of this review was to analyze studies that used a unique neuropsychological and psychological assessment in patients with NF1. Most of the literature studies have been conducted in child populations with NF1, because the pathology is more widespread. However, cognitive functions were not investigated by specific tests, but were measured through intelligence scales. A single case of an NF1 patient was assessed with a neuropsychological battery that included detailed cognitive domains with a follow-up evaluation [38]. In other studies, no changes in cognitive deficits from childhood to adulthood were described [32]. For this reason, these data were inconsistent in defining cognitive characteristics in these patients.

The findings we have just discussed in this review lead to the common conclusion that there is no standardized neuropsychological assessment for adults with NF1. However, the literature suggests its use because these patients show cognitive impairments that need to be considered. The challenge lies in obtaining a consensus on the battery of tests that would be the most reliable and useful for NF1 adults “efficiently and effectively” for “targeted pharmacological treatment and neuro-rehabilitative training” in the future. There are many tests for the evaluation of each cognitive domain, each differing in reliability, feasibility, and validity for specific populations. Most neuropsychological tests are designed for school-age children/adolescents, and there are few studies on the cognitive effects of aging in NF1. It is unclear which measures are most effective in characterizing cognitive functioning in older adults. Based on these considerations and on the literature research about cognitive domains that are more compromised in NF1, it is appropriate to give priority to the domain of attention and executive functions. A comprehensive assessment of all cognitive tests that can be utilized with NF1 is vital due to the severity of the impairments experienced by individuals with NF1, as well as for the development of targeted pharmacological and neuro-rehabilitative training to enhance these deficits. This article primarily focuses on the use of paper–pencil neuropsychological tools for adults and, based on the results obtained by concentrating only on IQ evaluations, it is conceivable that more specific tests for individual cognitive domains may be required. Specifically, the ROCF test has been used for visuospatial assessments, while the CORSI has been used for attentional evaluations. Additional research is required to scrutinize this aspect using other tests that assess the complexity of attentional processes, such as the Trail Making Test (TMT) and Visual Search. Additionally, no specific tests have been employed in the literature for executive functions. Hence, it would be beneficial to use a battery for the evaluation of the dysexecutive syndrome, comprising inhibitory control and cognitive flexibility, such as the Behavioral Assessment of the Dysexecutive Syndrome (BADS) and the Wisconsin Card Sorting Test (WCST). As previously mentioned, we included in our research two studies despite the NOS’s lower score (Table 1). In particular, Walsh et al. [26], because the study concerned the identification of standardized and specific cognitive assessment tools in NF clinical trials and was important to the problem of our review. Fjermestad et al. [27] did not use a standardized self-report survey. To date, in children, there is a combination of tests with a significant predictive value for NF1 about deficits in visual-spatial/motor abilities. This combination of tests that evaluate these domains could represent a strong discriminator of NF1; in fact, it was identified in 90% of individuals with the diagnosis [42]. This finding may be useful to provide evidence also in the adult population. Another reason why it would be useful to identify a unique neuropsychological assessment in these patients is the presence of cognitive impairment in elderly NF1 patients [43] and the association of dementia and NF1 [44]. Future studies could study the association of cognitive phenotype and NF1-related changes in brain structure in adults with NF1. Increased risk of dementia in NF1 may justify the consideration of neuropsychological testing and brain MRI in individuals with basic NF1 screening in clinical practice. It is important to realize that several limitations must be discussed. First of all, due to the fact that this research is based on published material, publication bias is an important factor. Furthermore, studies showed substantial heterogeneity in different areas such as population (number, age, gender, race, absence of follow-up, differences in mutation of NF1 genes). Second was the small number of longitudinal studies and evaluation in the adult population. Third, the evolution of cognitive capacities from adolescence to adulthood and high age seems to be a question that remains to be studied. Finally, this study was registered on the OSF platform after completion.

## 5. Conclusions

For future direction, there is a need for a large follow-up study from childhood to adulthood with a case–control design due to the evolution of cognitive profile during the entire life. As mentioned above, there was a correlation between cognitive dysfunction and T2 hyperintensity [19,20]. Therefore, a more in-depth analysis of biomarkers should be made, supported by the analysis of the neuropsychological profile in the future follow-up of clinical studies [45,46]. This review leads to the common conclusion that a battery of tests may be necessary for multicenter clinical NF1 trials. To reach a consensus, sensitive, validated, and reliable measures of cognitive outcome need to be selected and included in such a battery. Standardized methods would also serve cognitive rehabilitation purposes for cognitive impairment in persons with NF1 in the future.

## Figures and Tables

**Table 1:** Scores Newcastle–Ottawa Scale (NOS).

Article	Selection	Comparability	Outcome	Total Score
Struemph et al., 2021 [28]	3	1	2	6
Koini et al., 2017 [29]	3	1	2	6
Walsh et al., 2016 [26]	2	0	0	2
Schutze et al., 2018 [30]	3	1	2	6
Descheemaeker et al., 2013 [31]	4	2	2	8
Pavol et al., 2006 [32]	3	1	2	6
Bearden et al., 2015 [33]	4	1	3	8
Doser et al., 2022 [34]	4	2	2	8
Feldmann et al., 2003 [35]	4	2	2	8
Hellebrekers et al., 2022 [36]	4	1	2	7
Rowbotham et al., 2009 [37]	4	1	2	7
Miguel et al., 2015 [38]	2	0	3	5
Fjermestad et al., 2018 [27]	2	0	1	3
Vranceanu et al., 2016 [39]	4	1	3	8
Lester et al., 2020 [40]	4	2	3	9
Lester et al., 2023 [41]	4	1	2	7

**Table 2:** Characteristics of the studies included in the review.

Study	Participants	Inclusion Criteria	Cognitive Functions	Assessment Tools	Objective	**Results**
Feldmann R et al., 2003 [35]	100 patients with NF1 and 100 healthy control subjects matched for age, sex, and socioeconomic status	Matched for age and socioeconomic status.	IQ	WISC-R; WAIS-R.	To investigate if cognitive and motor difficulties in individuals with NF1 are linked to concentrated regions of heightened signal intensity (T2H).	Lower score in WAIS-R and fine motor skills
Pavol M et al., 2006 [32]	20 participants with NF-1 and 25 control participants (18 years or older)	Age: +18 years At least two of the following features: six or more light brown spots, lumps on or under the skin, freckling in armpits or groin, small bumps on the iris, bone abnormality, or a first-degree relative with NF-1.	Visual skills, attention, executive functions, and verbal abilities.	VMI; JLO; VFDT; BCT; PPVT–R; COWA.	To investigate decreased visuospatial and attention abilities.	Impairment on tests that use multiple cognitive skills.
Rowbotham I et al., 2009 [37]	16 NF1 patients and 16 age- and sex-matched controls	Matched for age and gender. Only subjects without any other diagnoses.	Perception, executive functioning (inhibitory control, cognitive flexibility, and working memory)	ANT	To identify variations in cognitive control that could be responsible in different cognitive areas or at different stages of processing information.	Deficits of reaction time in NF1 compared to controls
Descheemaeker MJ et al., 2013 [31]	20 NF1 adults and an IQ-, age-, and gender-matched control group (n = 20)	Age: +18 years Equal gender distribution and educational level.	Intelligence, visual-spatial abilities and memory, auditory memory, selective and sustained attention, and executive functioning	WAIS-III; ROCF; RAVLT; Bourdon–Wiersma; TMT; Stroop; COWA; ToL; WCST.	To examine neuropsychological traits that are associated with intellectual abilities.	Impairment of visual-spatial skills and auditory long-term memory in NF1 patients
Miguel CS et al., 2015 [38]	Case report of a 28-year-old, right-handed male, diagnosed with NF1 at the age of 10 years old.	-	Executive function, attention, verbal and visual memory, visuospatial function, and language	MMSE; WCST; WAIS-III; WAIS-R; Stroop; TMT, ROCF; CPT; COWA; RAVLT.	To examine the cognitive profile of an adult patient who has NF1 and cognitive pattern changes after a 14-month follow-up.	Visual memory, verbal learning, selective attention inhibitory control, and problem solving declined over time, whereas visual search, psychomotor speed, visuospatial function, and mental flexibility improved after 14 months.
Bearden CE et al., 2016 [33]	44 NF1 patients (n = 21 with placebo)	1. With NIH NF1 2. Young adults and adults 3. IQ of 70 or higher 4. Not taking statin medication 5. No hypercholesterolemia 6. Normal lab values 7. Not taking medication that may interact with lovastatin 8. No neurological or psychiatric disorder 9. No intracranial pathology except for asymptomatic optic pathway glioma 10. Women cannot be pregnant or lactating and must use adequate birth control measures 11. Fluent in English	Nonverbal declarative memory, working memory, attention, learning, social behavior, executive functions.	BVMT; LNS; D-CAT; HVLT; WISC-III; CBCL or YASR; NAB; D-KEFS; and BRIEF.	To assess lovastatin’s effects on cognition and behavior in patients with NF1	Beneficial effects of lovastatin on some learning and memory functions
Vranceanu AM et al., 2016 [39]	63 patients completed baseline assessments and were randomized.	Diagnosed with NF1, NF2, or schwannomatosis; age 18+; provide consent; read English at 6th-grade level; report stress with NF symptoms.	Quality of life, depression, anxiety	WHOQOL-BREF; PHQ-9; GAD-7.	To test the feasibility, acceptability, efficacy, and durability of a mind–body program vs. an attention placebo control both delivered via group videoconferencing.	Improvement of social relations and physical QoL, depression, anxiety, pain intensity.
Koini M et al., 2017 [29]	16 NF1 patients and 32 healthy controls	Young adults	Inhibitory control, verbal, and performance abilities.	WISC	To investigate the executive function related anterior thalamic radiation (ATR), cingulate bundle (CB), and superior longitudinal fasciculus (SLF) white matter— white matter integrity that can be considered as a pathological foundation for inhibitory control deficiencies in adolescents with NF1.	Damage to the anterior thalamic radiation related with inhibitory control
Fjermestad KW et al., 2018 [27]	142 persons with NF1 and 46293 controls from the HUNT3 population study	Matched for age.	Quality of life and activities of daily living	Self-report survey	To examine the HQoL problems among adults with NF1 and gender differences within the NF1 sample	NF1 sample reported significantly poorer life satisfaction, mental health, sleep disorders, more pain, gastrointestinal problems, and memory problems.
Schütze M et al., 2018 [30]	16 individuals diagnosed with NF1 and 16 non-psychiatric, non-neurologic, and non-oncologic individuals matched by age, education, and gender.	Young adult and adults Formal education: 2 to 16 years Socioeconomic status: low to middle	Intelligence, attention/processing speed, visuospatial abilities, episodic memory, fine motor coordination, and executive functions.	Brazilian WAIS-III, verbal (VIQ), procedure (PIQ) and full-scale (IQ) intelligence quotients; 9HPT; ROCF; RAVLT; VF; FDT; DGS and Corsi; ToL.	To explore correlation between cognitive abilities of NF1 patients and brain metabolism patterns.	Metabolic pattern relates to cognitive performance
Lester E et al., 2020 [40]	51 adolescents with NF were randomized.	Young adults diagnosed with NF1 or NF2; able to provide informed consent; English comprehension at a third-grade level; report stress and difficulties coping with NF symptoms.	Psychological quality of life (QoL), social relations QoL, environmental QoL, depression, anxiety, and pain interference	RY-NF	To examine the feasibility, acceptability, preliminary effect, and durability of a mind–body videoconferencing program for youth with neurofibromatosis against an experimental educational control.	Improvement of physical health QoL, psychological QoL, social relations QoL
Doser K et al., 2022 [34]	103 individuals with NF1	Born after 2 April 1968; did not have a tumour or tumour combinations; matched on sex, education, and employment status.	Intelligence; attentional set-shifting, planning and planning time, working memory, visual short-term memory, sustained attention and movement time and reaction time as well as visuospatial constructional ability and visuospatial memory; executive functions; autism spectrum disorder traits; quality of life; anxiety and depression.	WAIS-IV; MTT; OTS; SWM; SSP; RVP; ROCF; SRS-II; BRIEF-A; PedsQL developed for adults with NF1; PHQ-9; GAD-7.	To study the impacts of living with NF1 on health, socioeconomic status, and psychological well-being.	Impairment of quality of life and a high need for professional support for physical, psychological, and work-related problems
Hellebrekers DMJ et al., 2022 [36]	38 male patients with Duchanne Muscolar Distrophy were aged-matched with data of 38 male patients with NF1 (young adults)	(1) Dutch proficiency, (2) normal hearing, (3) no severe visual impairment, and (4) no physical immobility of upper limbs.	Intellectual abilities, sequential and simultaneous processing, verbal memory, and sustained attention.	WISC-III; KABC-II; RAVLT; TEA-Ch.	To evaluate variations in behavior and cognition between DMD and NF1	Low intellectual abilities, impairment of simultaneous processing, verbal memory, and sustained attention
Struemph KL et al., 2022 [28]	55 patients aged 16–34 years, with NF1.	Young adults and adults with pNF tumors.	Activities of daily living; verbal and perceptual reasoning abilities; overall intelligence score; processing speed; executive functioning; basic auditory attention and working memory; sustained attention.	VABS-II; WASI; WAIS-III; TMT and VF; DS; CPT II.	To explore the associations between adaptive functioning and cognitive factors.	Adaptive functioning and life achievement correlated with processing speed, executive functioning, and working memory scores
Lester EG et al., 2023 [41]	220 individuals with NF	Matched for age.	Depression, anxiety, perceived stress, pain, and general quality of life.	PHQ-9; GAD-7; PSS; GCPS-R; PROMIS; WHOQOL-BREF.	To examine severity and clinical correlates of suicidal ideation in adults with NF	Depression and poor psychological QoL significantly increased the risk for suicidal ideation.

**Legend:** WAIS = Wechsler Adult Intelligence Scale; 9HPT = Nine-Hole Peg Test; ROCF = Rey–Osterrieth Complex Figure Test; RAVLT = Rey Auditory Verbal Learning Test; VF = Verbal Fluency Test; FDT = The Five Digits Test; DGS = Digit Span; ToL = Tower of London Test; VABS = Vineland Adaptive Behavior Scales; WASI = Wechsler Abbreviated Scale of Intelligence; TMT = Trail Making Test; CPT = Conners Continuous Performance Test; WISC = Wechsler Intelligence Scale for Children; MTT = Multitasking Test; OTS = One-touch Stocking of Cambridge; SWM = Spatial Working Memory; SSP = Spatial Span; RVP = Rapid Visual Information Processing; SRS = Social Responsiveness Scale; BRIEF-A = Behavior Rating Inventory of Executive Function—Adult Version; PedsQL = Pediatric Quality of Life Inventory; PHQ-9 = Patient Health Questionnaire; GAD-7 = Seven-item Generalized Anxiety Disorder scale; COWA = Controlled Oral Word Association Test; WCST = Wisconsin Card Sorting Test; MMSE = Mini-Mental State Exam; WCST = Wisconsin Card Sorting Test; CPT = Continuous Performance Test; ANT = Amsterdam Neuropsychological Tasks; KABC = Kaufmann Assessment Battery for Children; TEA-Ch = Test of Everyday Attention for Children; WISC-R = Wechsler Intelligence Scale for Children—Revised; WAIS-R = Wechsler Adult Intelligence Scale—Revised; BVMT = Brief Visuospatial Memory Test; LNS = Letter-Number Sequencing task; D-CAT = Digit Cancellation test; HVLT = Hopkins Verbal Learning Test; CBCL = Achenbach Child Behavior Checklist; YASR = Young Adult Self Report; NAB = Neuropsychological Assessment Battery; D-KEFS = Delis–Kaplan Executive Function Scale; VMI = Developmental Test of Visual-Motor Integration; JLO = Judgment of Line Orientation; VFDT = Visual Form Discrimination; BCT = Booklet Category Test; PPVT–R = Peabody Picture Vocabulary Test—Revised; WHOQOL-BREF = World Health Organization QoL Abbreviated Instrument; PSS = Perceived Stress Scale; GCPS-R = Graded Chronic Pain Scale; PROMIS = Pain Interference Short Form; RY-NF = Resilient Youth with NF.

## References

[1] Williams V.C., Lucas J., Babcock M.A., Gutmann D.H., Korf B., Maria B.L. (2009). Neurofibromatosis type 1 revisited. Pediatrics.

[2] Iheanacho I., Yoo H.K., Yang X., Dodman S., Hughes R., Amin S. (2022). Epidemiological and clinical burden associated with plexiform neurofibromas in pediatric neurofibromatosis type-1 (NF-1): A systematic literature review. Neurol. Sci..

[3] Upadhyaya M., Ruggieri M., Maynard J., Osborn M., Hartog C., Mudd S., Penttinen M., Cordeiro I., Ponder M., Ponder B.A.J. (1998). Gross deletions of the neurofibromatosis type 1 (NF1) gene are predominantly of maternal origin and commonly associated with a learning disability, dysmorphic features and developmental delay. Hum. Genet..

[4] Tucker T., Wolkenstein P., Revuz J., Zeller J., Friedman J.M. (2005). Association between benign and malignant peripheral nerve sheath tumors in NF1. Neurology.

[5] Pilavaki M., Chourmouzi D., Kiziridou A., Skordalaki A., Zarampoukas T., Drevelengas A. (2004). Imaging of peripheral nerve sheath tumors with pathologic correlation: Pictorial review. Eur. J. Radiol..

[6] Zhu Y., Ghosh P., Charnay P., Burns D.K., Parada L.F., Power S., Lengaigne M., Saitou M., Hayashi K., Swaney D.L. (2002). Neurofibromas in NF1: Schwann cell origin and role of tumor environment. Science.

[7] Carroll S.L., Ratner N. (2008). How does the Schwann cell lineage form tumors in NF1?. Glia.

[8] Chisholm A.K., Haebich K.M., Pride N.A., Walsh K.S., Lami F., Ure A., Maloof T., Brignell A., Rouel M., Granader Y. (2022). Delineating the autistic phenotype in children with neurofibromatosis type 1. Mol. Autism.

[9] Potvin D., Hardy K.K., Walsh K.S. (2015). The relation between ADHD and cognitive profiles of children with NF1. J. Pediatr. Neuropsychol..

[10] A Champion J., Rose K.J., Payne J.M., Burns J., North K.N. (2014). Relationship between cognitive dysfunction, gait, and motor impairment in children and adolescents with neurofibromatosis type 1. Dev. Med. Child Neurol..

[11] Payne J.M., Haebich K.M., MacKenzie R., Walsh K.S., Hearps S.J.C., Coghill D., Barton B., Pride N.A., Ullrich N.J., Tonsgard J.H. (2021). Cognition, ADHD Symptoms, and Functional Impairment in Children and Adolescents with Neurofibromatosis Type 1. J. Atten. Disord..

[12] Nupan M.M.T., Van Meerbeke A.V., Cabra C.A.L., Gomez P.M.H. (2017). Cognitive and Behavioral Disorders in Children with Neurofibromatosis Type 1. Front. Pediatr..

[13] Isenberg J.C., Templer A., Gao F., Titus J.B., Gutmann D.H. (2013). attention skills in children with neurofibromatosis type 1. J. Child Neurol..

[14] Hyman S.L., Shores A., North K.N. (2005). The nature and frequency of cognitive deficits in children with neurofibromatosis type 1. Neurology.

[15] Beaussart M.-L., Barbarot S., Mauger C., Roy A. (2018). Systematic Review and Meta-analysis of Executive Functions in Preschool and School-Age Children with Neurofibromatosis Type 1. J. Int. Neuropsychol. Soc..

[16] Lehtonen A., Howie E., Trump D., Huson S.M. (2013). Behaviour in children with neurofibromatosis type 1: Cognition, executive function, attention, emotion, and social competence. Dev. Med. Child Neurol..

[17] Hyman S., Gill D., Shores E., Steinberg A., Joy P., Gibikote S., North K. (2003). Natural history of cognitive deficits and their relationship to MRI T2-hyperintensities in NF1. Neurology.

[18] Piscitelli O., Digilio M.C., Capolino R., Longo D., Di Ciommo V. (2012). Neurofibromatosis type 1 and cerebellar T2-hyperintensities: The relationship to cognitive functioning. Dev. Med. Child Neurol..

[19] Molinari M., Petrosini L., Misciagna S., Leggio M.G. (2004). Visuospatial abilities in cerebellar disorders. J. Neurol. Neurosurg. Psychiatry.

[20] Hyman S.L., Gill D.S., Shores E.A., Steinberg A., North K.N. (2007). T2 hyperintensities in children with neurofibromatosis type 1 and their relationship to cognitive functioning. J. Neurol. Neurosurg. Psychiatry.

[21] Puente A., Geisinger K.F., Bracken B.A., Carlson J.F., Hansen J.-I.C., Kuncel N.R., Reise S.P., Rodriguez M.C. (2013). Assessment of neuropsychological functioning. APA Handbook of Testing and Assessment in Psychology.

[22] Lezak M.D., Howieson D.B., Loring D.W. (2004). Neuropsychological Assessment.

[23] Meier M.J., Maruish M.E., Moses J.A. (1997). The establishment of clinical neuropsychology as a specialty. Clinical Neuropsychology: Theoretical Foundations for Practitioners.

[24] Tonkonogy J., Puente A.E. (2009). Localization of Clinical Syndromes in Neuropsychology and Neuroscience.

[25] Wells G.A., Shea B., O’Connell D., Peterson J., Welch V., Losos M., Tugwell P. The Newcastle-Ottawa Scale (NOS) for Assessing the Quality of Nonrandomised Studies in Meta-Analyses. https://www.ohri.ca/programs/clinical_epidemiology/oxford.asp.

[26] Walsh K.S., Janusz J., Wolters P.L., Martin S., Klein-Tasman B.P., Toledo-Tamula M.A., Thompson H.L., Payne J.M., Hardy K.K., de Blank P. (2016). Neurocognitive outcomes in neurofibromatosis clinical trials: Recommendations for the domain of at-tention. Neurology.

[27] Fjermestad K.W., Nyhus L., Kanavin J., Heiberg A., Hoxmark L.B. (2018). Health Survey of Adults with Neurofibromatosis 1 Compared to Population Study Controls. J. Genet. Couns..

[28] Struemph K.L., Watts A.T.M., Wolters P.L., Tamula M.A., Baldwin A., Widemann B., Martin S. (2022). Adolescents and young adults with neurofibromatosis type 1: A descriptive study of adaptive functioning. Am. J. Med. Genet. Part A.

[29] Koini M., Rombouts S.A.R.B., Veer I.M., Van Buchem M.A., Huijbregts S.C.J. (2017). White matter microstructure of patients with neurofibromatosis type 1 and its relation to inhibitory control. Brain Imaging Behav..

[30] Schütze M., Costa D.d.S., de Paula J.J., Malloy-Diniz L.F., Malamut C., Mamede M., de Miranda D.M., Brammer M., Romano-Silva M.A. (2018). Use of machine learning to predict cognitive performance based on brain metabolism in Neurofibromatosis type 1. PLoS ONE.

[31] Descheemaeker M., Plasschaert E., Frijns J., Legius E. (2013). Neuropsychological profile in adults with neurofibromatosis type 1 compared to a control group. J. Intellect. Disabil. Res..

[32] Pavol M., Hiscock M., Massman P., Moore I.B., Foorman B., Meyers C. (2006). Neuropsychological function in adults with von recklinghausen’s neurofibromatosis. Dev. Neuropsychol..

[33] Bearden C.E., Hellemann G.S., Rosser T., Montojo C., Jonas R., Enrique N., Pacheco L., Hussain S.A., Wu J.Y., Ho J.S. (2016). A randomized placebo-controlled lovastatin trial for neurobehavioral function in neurofibromatosis I. Ann. Clin. Transl. Neurol..

[34] Doser K., Hove H., Østergaard J.R., E Bidstrup P., O Dalton S., Handrup M.M., Ejerskov C., Krøyer A., Doherty M.A., Jepsen J.R.M. (2022). Cohort profile: Life with neurofibromatosis 1—The Danish NF1 cohort. BMJ Open.

[35] Feldmann R., Denecke J., Grenzebach M., Schuierer G., Weglage J. (2003). Neurofibromatosis type 1: Motor and cognitive function and T2-weighted MRI hyperintensities. Neurology.

[36] Hellebrekers D.M.J., van Abeelen S.A.M., Catsman C.E., van Kuijk S.M.J., Laridon A.M., Klinkenberg S., Hendriksen J.G.M., Vles J.S.H. (2022). Cognitive and behavioral functioning in two neurogenetic disorders; how different are these aspects in Duchenne muscular dystrophy and Neurofibromatosis type 1?. PLoS ONE.

[37] Rowbotham I., Cate I.M.P.-T., Sonuga-Barke E.J.S., Huijbregts S.C.J. (2009). Cognitive control in adolescents with neurofibromatosis type 1. Neuropsychology.

[38] Miguel C., Chaim T., Silva M.A., Louzã M.R. (2015). Neurofibromatosis type 1 and attention deficit hyperactivity disorder: A case study and literature review. Neuropsychiatr. Dis. Treat..

[39] Vranceanu A.-M., Riklin E., Merker V.L., Macklin E.A., Park E.R., Plotkin S.R. (2016). Mind–body therapy via videoconferencing in patients with neurofibromatosis. Neurology.

[40] Lester E., DiStefano S., Mace R., Macklin E., Plotkin S., Vranceanu A.-M. (2020). Virtual mind-body treatment for geographically diverse youth with neurofibromatosis: A pilot randomized controlled trial. Gen. Hosp. Psychiatry.

[41] Lester E.G., Wang K.E.B., Blakeley J.O., Vranceanu A.-M. (2023). Occurrence and Severity of Suicidal Ideation in Adults with Neurofibromatosis Participating in a Mind–Body RCT. Cogn. Behav. Neurol..

[42] Schrimsher G.W., Billingsley R.L., Slopis J.M., Moore B.D. (2003). Visual-spatial performance deficits in children with neurofibromatosis type-1. Am. J. Med. Genet. A.

[43] Costa Dde S., de Paula J.J., de Rezende N.A., Rodrigues L.O., Malloy-Diniz L.F., Romano-Silva M.A., Miranda D.M. (2014). Neuropsychological impairments in elderly Neurofibromatosis type 1 patients. Eur. J. Med. Genet..

[44] Kallionpää R.A., Valtanen M., Auranen K., Uusitalo E., Rinne J.O., Peltonen S., Peltonen J. (2021). Increased risk for dementia in neurofibromatosis type 1. Genet. Med..

[45] Baudou E., Nemmi F., Biotteau M., Maziero S., Peran P., Chaix Y. (2020). Can the cognitive phenotpe in neurofibomatosis type 1 (NF1) be explained by neuroimaging? A review. Front. Neurol..

[46] Duarte J.V., Ribeiro M.J., Violante I.R., Cunha G., Silva E., Castelo-Branco M. (2014). Multivariate pattern analysis reveals subtle brain anomalies relevant to the cognitive phenotype in neurofibromatosis type 1. Human Brain Mapp..

